# Machine Learning-Based Prediction of Sleep Quality in Patients with Multiple Sclerosis

**DOI:** 10.3390/medsci14040483

**Published:** 2026-08-14

**Authors:** Laura-Elena Cucu, Laura-Cristina Baciu, Oriana-Maria Onicescu, Bogdan-Emilian Ignat, Alina Săcărescu, Andra Oancea, Cristina Grosu, Costin Chirica, Gabriela Popescu, Alexandra Maștaleru, Robert-Valentin Bîlcu, Andreea Mustață, Mihai Roca, Maria-Magdalena Leon

**Affiliations:** 1 Doctoral School, “Grigore T. Popa” University of Medicine and Pharmacy, 700115 Iasi, Romania; dudau.laura-elena@d.umfiasi.ro (L.-E.C.); chirica.costin@d.umfiasi.ro (C.C.); robert-valentin.bilcu@d.umfiasi.ro (R.-V.B.); 2 Clinical Rehabilitation Hospital, 700661 Iasi, Romania; emilian.ignat@umfiasi.ro (B.-E.I.); alina.sacarescu1@umfiasi.ro (A.S.); andra.oancea@umfiasi.ro (A.O.); cristina.grosu@umfiasi.ro (C.G.); gabriela.popescu@umfiasi.ro (G.P.); alexandra.mastaleru@umfiasi.ro (A.M.); m_andreea1998@icloud.com (A.M.); roca2m@yahoo.com (M.R.); maria.leon@umfiasi.ro (M.-M.L.); 3 Faculty of Computer Science, Alexandru Ioan Cuza University Iasi, 700506 Iasi, Romania; oriana.oniciuc@info.uaic.ro; 4 Department of Neurology, “Grigore T. Popa” University of Medicine and Pharmacy, 700115 Iasi, Romania; 5 Department of Medical Specialties I, “Grigore T. Popa” University of Medicine and Pharmacy, 700115 Iasi, Romania

**Keywords:** multiple sclerosis, sleep quality, Pittsburgh Sleep Quality Index, machine learning, SHAP, restless legs syndrome

## Abstract

**Background/Objectives**: Sleep disturbances are common but frequently underrecognized in multiple sclerosis (MS), independently predicting reduced quality of life and worsening fatigue, cognitive impairment, and depression. While pain, nocturia, fatigue, and mood symptoms are established contributors, cardiometabolic, and inflammatory factors remain largely unexplored despite their known links to poor sleep in other populations. This study used interpretable machine learning to determine the relative contribution of disease-related, cardiometabolic, and inflammatory parameters to sleep quality, assessed by the PSQI, in patients with MS. **Methods**: This cross-sectional, observational, single-center cohort study enrolled adult patients with MS. Sleep quality (PSQI), daytime sleepiness (ESS), and restless legs syndrome severity (IRLS) were assessed alongside clinical, anthropometric, hemodynamic, and laboratory parameters, including inflammatory and metabolic indices. Three regression models, Support Vector Regression (SVR), Random Forest, and XGBoost, were trained on 28 predictors to predict PSQI global scores and evaluated on an internal test set, with feature contributions examined using SHAP analysis. **Results**: A total of 173 patients with MS were included (mean age 39.66 ± 11.86 years, 69.9% female, 90.2% RRMS), with 48.0% classified as poor sleepers (PSQI > 5) and a mean PSQI score of 6.06 ± 3.47. XGBoost achieved the best predictive performance (test R^2^ = 0.451). SHAP analysis identified IRLS severity as the strongest predictor of PSQI across all three models, followed by EDSS score and depression in the Random Forest and XGBoost models, while daytime sleepiness (ESS) ranked consistently among the top predictors. Cardiometabolic and inflammatory parameters contributed inconsistently, with several showing effects opposite to physiological expectation. **Conclusions**: Sleep impairment in MS was most strongly associated with restless legs syndrome severity and neurological disability, with depression also contributing in the two best-performing models. Disease-related and symptomatic factors outweighed cardiometabolic and inflammatory contributions. These findings support the value of interpretable machine learning for identifying clinically relevant correlates of sleep quality in MS, though the exploratory design and modest sample size warrant confirmation in larger, independent cohorts.

## 1. Introduction

Multiple sclerosis (MS) is a chronic, immune-mediated disease of the central nervous system characterized by inflammation, demyelination, and progressive neurodegeneration [1]. It represents one of the leading causes of non-traumatic neurological disability in young adults, typically manifesting between the second and fourth decades of life and showing a clear female predominance [2]. Beyond the hallmark neurological deficits, patients with MS frequently experience a broad spectrum of non-motor symptoms that substantially affect daily functioning [3].

Among these non-motor manifestations, sleep disturbances are among the most prevalent yet frequently underrecognized, encompassing insomnia, restless legs syndrome (RLS), and excessive daytime sleepiness [4]. Sleep quality is significantly worse in MS than in healthy controls [5], and within this population poor sleep independently predicts reduced physical and mental health-related quality of life [6]. These disturbances are clinically consequential, contributing to fatigue, cognitive impairment, and mood disorders, thereby compounding the overall disease burden [7,8]. Importantly, sleep quality is a potentially modifiable determinant of the clinical course and outcomes of MS, making it a relevant target for assessment and intervention [9,10].

Despite their high prevalence and clinical impact, sleep disturbances in MS remain insufficiently characterized and are often overlooked in routine clinical assessment [11].

Sleep disturbance in MS has a multifactorial basis, with pain, nocturia, fatigue, and mood symptoms all implicated [12]. Beyond these established contributors, cardiometabolic and inflammatory factors may also play a role, as both have been linked to poor sleep in other populations [13,14,15]. Given that inflammation is intrinsic to MS and cardiometabolic dysfunction is a frequent comorbidity [16], their relative contribution to sleep quality in this population warrants closer examination.

To address this, we investigated the relative contribution of disease-related, cardiometabolic and inflammatory factors to sleep quality in MS using machine learning. By integrating clinical, neurological, metabolic, and inflammatory parameters into interpretable predictive models, we sought to identify which factors most strongly shape sleep quality. Because sleep disturbance does not follow a strictly linear pattern, traditional linear regressions are limited in capturing how these parameters interact. Machine learning algorithms are well-suited to handle these non-linear relationships and highly correlated clinical data [17]. These algorithms have been increasingly applied in MS to predict outcomes such as neuroaxonal injury [18] and quality of life [19], yet to our knowledge no study has used it to predict sleep quality, as assessed by the Pittsburgh Sleep Quality Index (PSQI), in this population.

## 2. Materials and Methods

### 2.1. Study Design and Population

This was a cross-sectional, observational, single-center cohort study conducted between December 2025 and June 2026. The study was conducted in accordance with the Declaration of Helsinki and was approved by the Ethics Committee of the Clinical Rehabilitation Hospital (Approval No. 19; date of approval: 16 October 2025) and by the Research Ethics Committee of the University of Medicine and Pharmacy “Grigore T. Popa” Iași (Approval No. 676; date of approval: 12 November 2025). Written informed consent was obtained from all participants prior to enrollment.

The study population comprised adult patients with a confirmed diagnosis of multiple sclerosis (MS), established according to the revised 2017 McDonald criteria [20]. Inclusion criteria were: (1) age of 18 years or older; (2) clinical stability at the time of enrollment, defined as the absence of relapse or corticosteroid treatment within the four weeks preceding assessment; and (3) ability to provide written informed consent and complete self-administered questionnaires.

Exclusion criteria were: (1) pregnancy; (2) presence of other significant neurological disorders; (3) comorbid rheumatological conditions; (4) major psychiatric disorders.

Clinical and demographic data collected at baseline included: age, sex, disease phenotype, disease duration, number of relapses, depression diagnosis, Expanded Disability Status Scale (EDSS) score, and current disease-modifying therapy (DMT). Depression was defined as an active clinical diagnosis and/or ongoing antidepressant treatment at the time of enrollment. DMTs were classified into moderate-efficacy (ME-DMTs: interferons, glatiramer acetate, dimethyl fumarate, teriflunomide) and high-efficacy (HE-DMTs: fingolimod, natalizumab, ocrelizumab, ofatumumab, alemtuzumab, cladribine) categories, following Delphi Consensus criteria [21,22]. All symptomatic medications were recorded, including gabapentinoids, baclofen, antihypertensives, statins, and pharmacological sleep aids.

### 2.2. Clinical Assessment and Data Collection

All data were collected during a single clinical assessment, including questionnaires, neurological examination, anthropometric and hemodynamic measurements, and laboratory investigations.

Questionnaires: Sleep quality was assessed using PSQI [23], a validated 19-item instrument yielding a global score from 0 to 21, with scores above 5 indicating clinically significant sleep disturbance. Daytime sleepiness was evaluated with the Epworth Sleepiness Scale (ESS) [24], with scores above 10 indicating excessive daytime sleepiness. Restless legs syndrome was diagnosed through a clinical interview conducted by the principal investigator (L.-E.C.), applying the diagnostic criteria of the International Restless Legs Syndrome Study Group (IRLSSG), updated in 2014 [25]. Patients meeting these criteria were then assessed for severity using the International Restless Legs Syndrome Scale (IRLS) [26]. Patients not meeting the IRLSSG criteria were assigned an IRLS score of 0.

Neurological examination: Disability was assessed using EDSS [27].

Anthropometric and hemodynamic measurements: Body weight was measured using a calibrated mechanical scale, and height using a stadiometer, with participants in light clothing and without shoes; BMI was calculated as weight (kg)/height^2^ (m^2^). Waist circumference was measured using a flexible tape at the midpoint between the lower costal margin and the iliac crest. Blood pressure and heart rate were measured after 5 min of seated rest, with blood pressure measured three times at 1 min intervals and the average of the last two readings recorded.

Laboratory investigations: Fasting laboratory parameters assessed at baseline (following a minimum of 8 h of fasting) included Erythrocyte Sedimentation Rate (ESR), uric acid, lipid profile, fasting glucose, ferritin, and complete blood count, obtained from routine venous blood samples processed on the same day at the hospital laboratory. The neutrophil-to-lymphocyte ratio (NLR) and systemic immune-inflammation index (SII = platelet count × NLR) were derived.

### 2.3. Statistical Analysis

Descriptive statistics were calculated to summarize participant characteristics. Continuous variables are presented as mean ± standard deviation (SD) or median and interquartile range (IQR), depending on data distribution, assessed using the Shapiro–Wilk test. Categorical variables are reported as counts (n) and percentages (%). All statistical analyses and model development were performed in Python version 3.13.5, using the following libraries: pandas 3.0.5, matplotlib 3.11.1, seaborn 0.13.2, scipy 1.18.0, scikit-learn 1.9.0, XGBoost 3.4.0, and SHAP 0.52.0.

### 2.4. Machine Learning Models

To predict PSQI global scores, three regression models were developed and compared: Support Vector Regression (SVR) with a linear kernel, Random Forest Regressor, and XGBoost Regressor. The dataset was split into training (80%, n = 138) and test (20%, n = 35) sets. A preprocessing pipeline was constructed using a ColumnTransformer with standard scaling for continuous features, and one-hot encoding for categorical variables. Hyperparameter tuning was performed for each model using GridSearchCV with 5-fold cross-validation on the training set, with R^2^ as the optimization metric. Final models were then evaluated on the held-out test set drawn from the same cohort.

The random seed used in all experiments was 42. The search space was defined as follows: SVR was evaluated over kernels {linear, RBF}, regularization parameter C ∈ {0.1, 1, 10}, kernel coefficient γ ∈ {scale, auto}, and ε-insensitive tube width ε ∈ {0.1, 0.2, 0.5}; Random Forest was tuned over the number of trees {50, 100, 150}, maximum depth {2, 3, 5}, and minimum samples required for a split {2, 5}; XGBoost was optimized with respect to the number of boosting rounds {50, 100, 150}, maximum tree depth {2, 3, 5}, learning rate {0.01, 0.1, 0.2}, and column subsampling ratio {0.5, 0.75, 1.0}. Missing values were imputed using a K-Nearest Neighbors (KNN) imputer (k = 5) for continuous features and a most-frequent strategy for categorical features, followed by one-hot encoding of categorical variables. Imputation, scaling, and encoding were performed separately within each fold to prevent data leakage.

The final feature set comprised 28 predictors organized into two domains: 

The disease-related and symptomatic domain included: MS phenotype, EDSS score, IRLS score, disease duration, number of relapses, DMT category, ESS score, gabapentinoid use (daytime and evening), baclofen use (daytime and evening), and depression. 

The cardiometabolic and inflammatory domain included: smoking status, coffee consumption, sex, waist circumference, Body Mass Index (BMI), systolic and diastolic blood pressure, High-Density Lipoprotein (HDL) cholesterol, uric acid, triglycerides, fasting glucose, diabetes, ferritin, ESR, NLR, SII.

Model performance was evaluated using the coefficient of determination (R^2^), Mean Absolute Error (MAE), and Root Mean Squared Error (RMSE). Model interpretability was assessed using SHAP (SHapley Additive exPlanations) analysis, with TreeExplainer applied to the Random Forest and XGBoost models and KernelExplainer to the SVR.

## 3. Results

### 3.1. Baseline Characteristics of the Study Population

The study included 173 patients with MS, predominantly female (69.9%) and with a mean age of 39.66 ± 11.86 years. The majority had relapsing–remitting MS (RRMS) (90.2%), with a mean disease duration of 10.84 ± 7.61 years and a mean EDSS score of 2.73 ± 1.76.

Sleep disturbance was prevalent in this cohort, with nearly half of participants (48.0%) classified as poor sleepers (PSQI > 5), and a mean PSQI global score of 6.06 ± 3.47. Restless legs syndrome was present in 76 patients (43.9%), with a median IRLS severity score of 16.0 (95% CI: 13.0–19.5) among affected individuals. Comorbidities included dyslipidemia (17.3%), hypertension (12.7%), depression (12.1%), and diabetes (3.5%, all type 2 diabetes). A complete summary of baseline characteristics is presented in Table 1.

The overall proportion of missing data across all predictors was 2%, with the highest rate observed for coffee consumption and number of relapses (11%). The missing data for each predictor are reported in Supplementary Table S1.

The correlation matrix (Supplementary Figure S1) showed that disease-related variables were more strongly correlated with PSQI than cardiometabolic and inflammatory markers.

### 3.2. Machine Learning Model Selection and Performance

The selected optimal configurations were: SVR with a linear kernel (C = 0.1, γ = scale, ε = 0.5); Random Forest with 50 trees, maximum depth of 5, and minimum samples per split of 5; and XGBoost with 50 estimators, maximum depth of 2, learning rate of 0.2, and column subsampling ratio of 0.5. The prevalence of shallow trees (max_depth ≤ 5) and conservative regularization across all three models reflects the small sample size (n = 173) and high dimensionality of the feature space, indicating that the models prioritized generalization over complexity to mitigate overfitting.

The performance metrics for all three regression models are summarized in Table 2. XGBoost achieved the highest test R^2^ (0.451), MAE of 1.956, and RMSE of 2.392. Random Forest yielded a test R^2^ of 0.380 (MAE = 2.138, RMSE = 2.542), while SVR showed the lowest test R^2^ of 0.336 (MAE = 2.322, RMSE = 2.630). Training R^2^ values were substantially higher for Random Forest (0.734) and XGBoost (0.864) compared to SVR (0.376). A reference model predicting the mean PSQI value showed a test R^2^ of −0.059, MAE of 2.880, and RMSE of 3.322.

**Figure 1:**
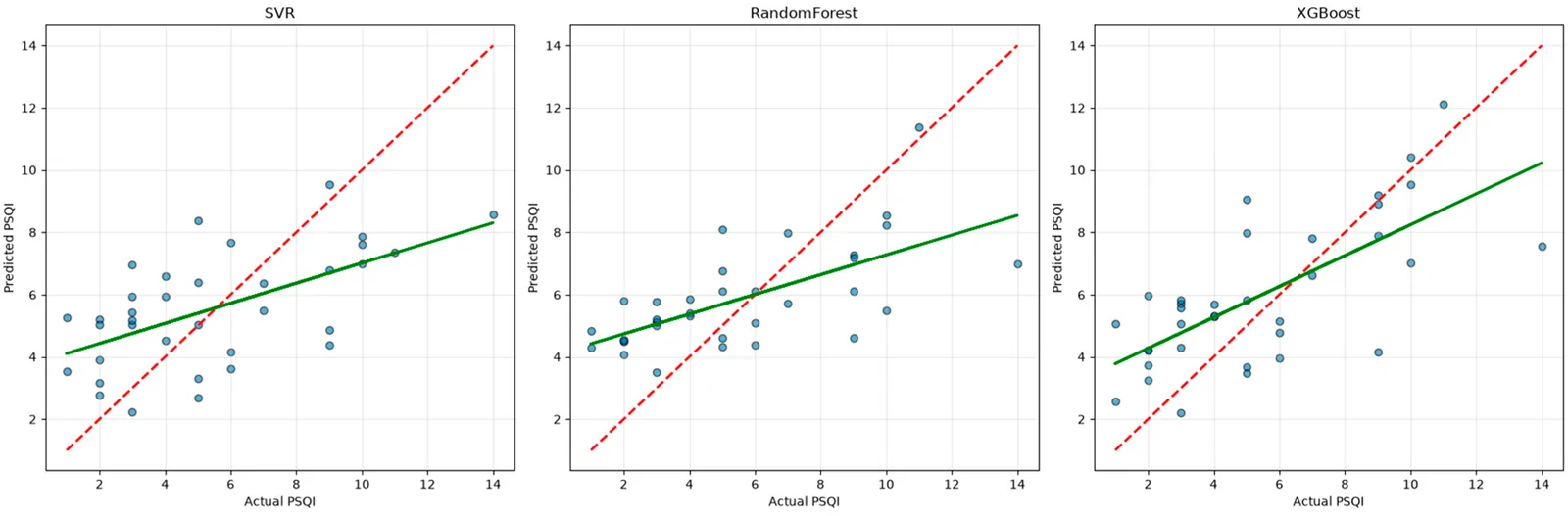
Observed versus predicted PSQI scores on the test set for all three models. The dashed line indicates where predicted values would equal observed values. The solid line indicates the linear trend.

**Figure 2:**
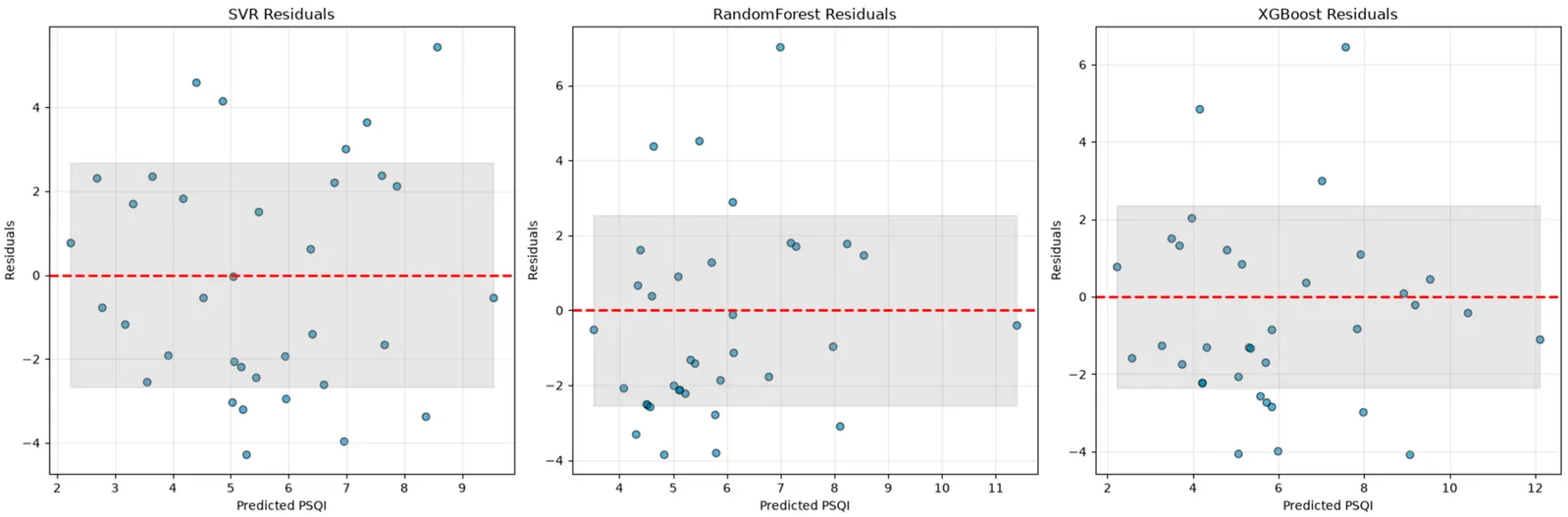
Residual plots (observed minus predicted PSQI) for SVR, Random Forest, and XGBoost models on the test set. The dashed red line indicates perfect prediction (residual = 0). The shaded area represents ± 1 standard deviation of the residuals.

Observed-versus-predicted and residual plots for all three models are presented in Figure 1 and Figure 2. Predicted PSQI values remained within the theoretical 0–21 range for all three models, ranging from 2.22 to 9.54 (SVR), 3.51 to 11.38 (Random Forest), and 2.21 to 12.10 (XGBoost), compared with an actual range of 1.00 to 14.00 in the test set.

### 3.3. Feature Importance and SHAP Analysis

**Figure 3:**
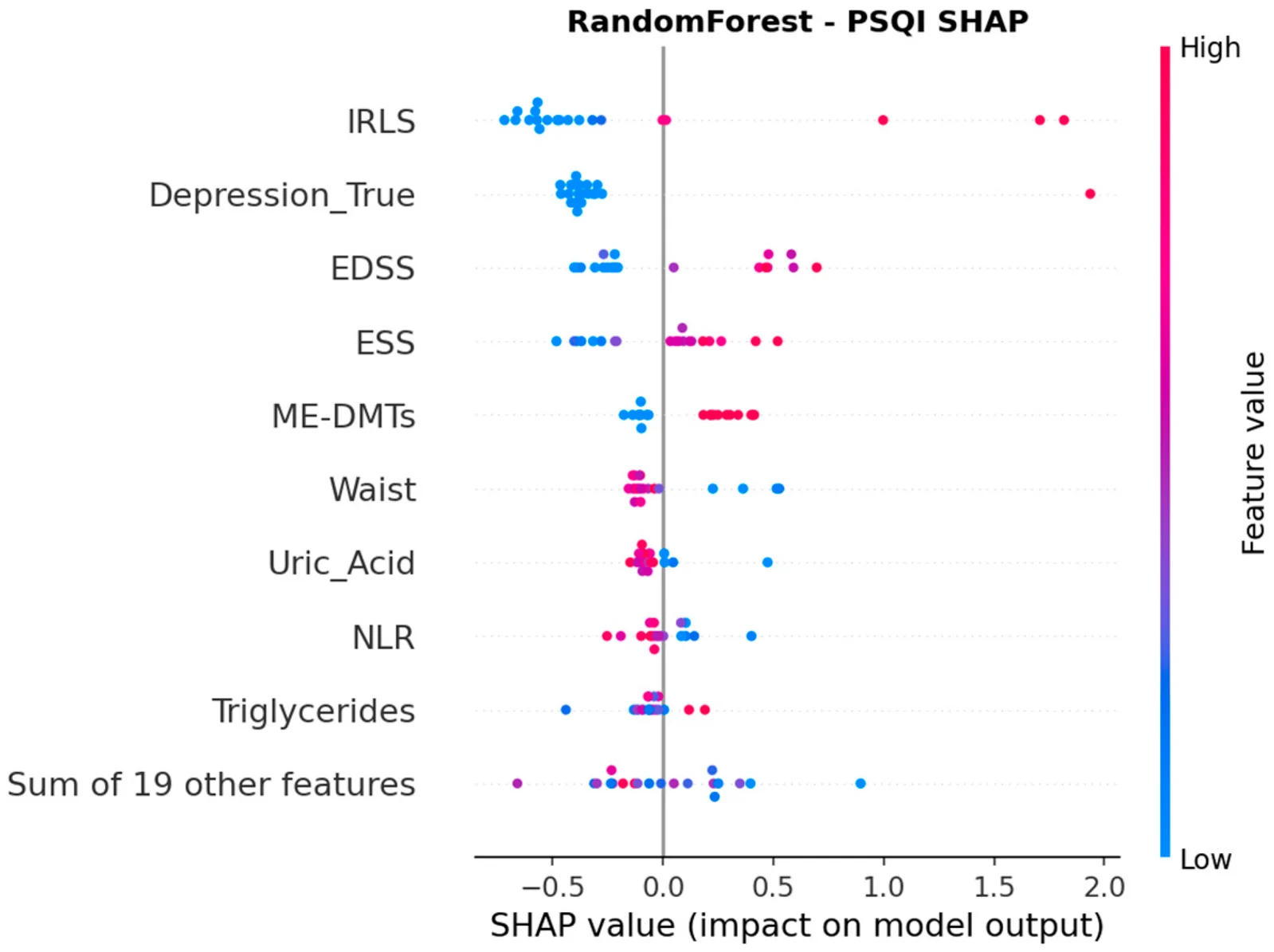
SHAP beeswarm summary plot for the Random Forest model. Features are ranked by mean absolute SHAP value. Positive values indicate a contribution toward higher predicted PSQI scores. Abbreviations: IRLS—International Restless Legs Syndrome Scale score; EDSS—Expanded Disability Status Scale score; ESS—Epworth Sleepiness Scale score; ME-DMTs—moderate-efficacy disease-modifying therapies; Waist—waist circumference; NLR—neutrophil-to-lymphocyte ratio.

In the Random Forest model (Figure 3), the top five predictors were IRLS, depression, EDSS, ESS, and ME-DMT treatment category. Higher IRLS, EDSS, and ESS scores, as well as the presence of depression and ME-DMT treatment, were associated with positive SHAP values, indicating a contribution toward higher predicted PSQI scores. Waist circumference, uric acid, NLR, and triglycerides were also identified among the top predictors, with higher values associated with negative SHAP values.

**Figure 4:**
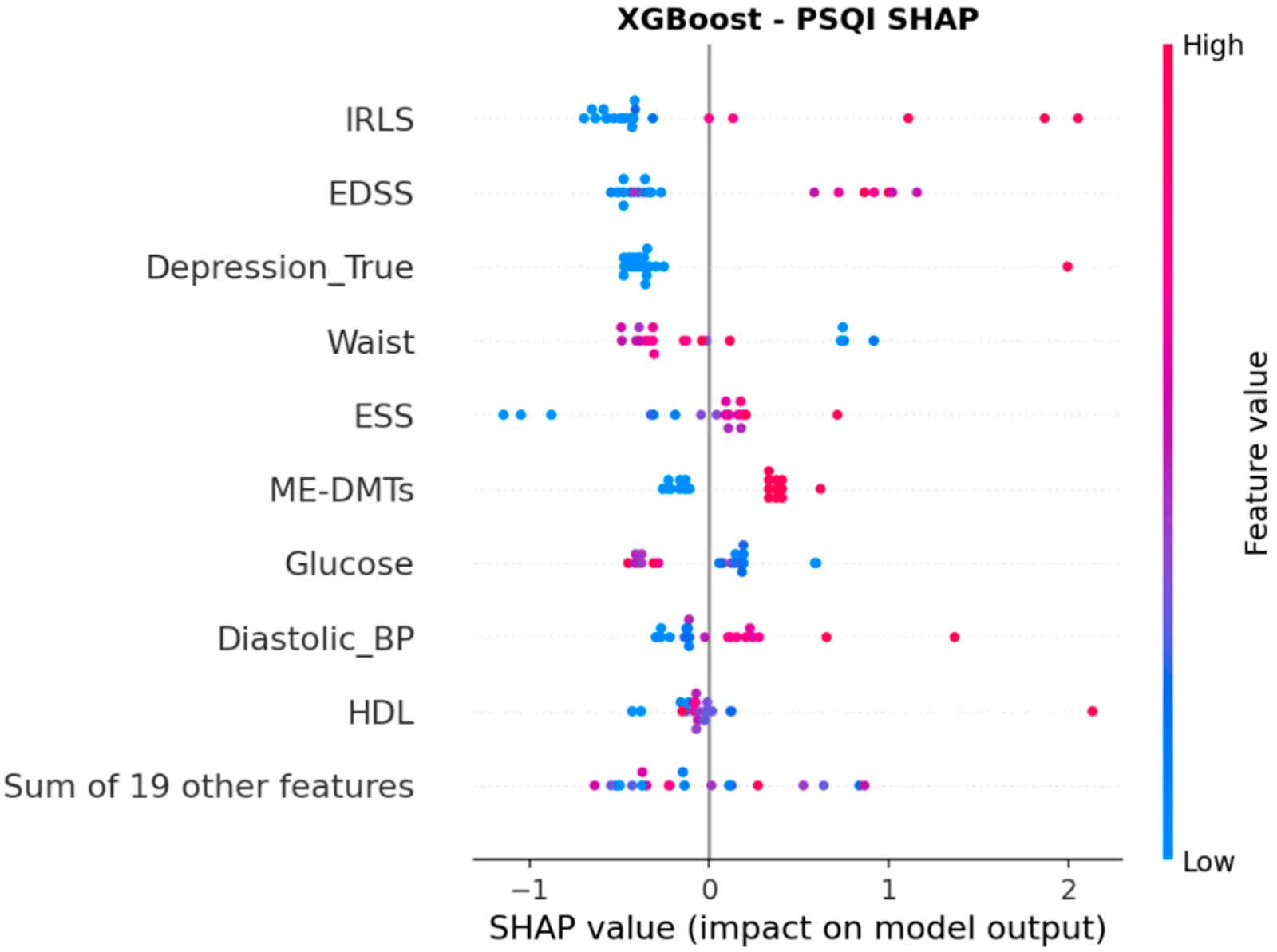
SHAP beeswarm summary plot for the XGBoost model. Features are ranked by mean absolute SHAP value. Positive values indicate a contribution toward higher predicted PSQI scores. Abbreviations: IRLS—International Restless Legs Syndrome Scale score; EDSS—Expanded Disability Status Scale score; Waist—waist circumference; ESS—Epworth Sleepiness Scale score; ME-DMTs—moderate-efficacy disease-modifying therapies; Diastolic_BP—diastolic blood pressure; HDL—high-density lipoprotein cholesterol.

In the XGBoost model (Figure 4), IRLS, EDSS, and depression were the top three predictors, with higher values, and the presence of depression, associated with positive SHAP values, indicating a contribution toward higher predicted PSQI scores. Larger waist circumference was associated with negative SHAP values, while higher ESS scores contributed toward higher predicted scores. ME-DMT treatment category, fasting glucose, diastolic blood pressure, and HDL-cholesterol were also identified among the top predictors, with higher glucose values associated with negative SHAP values and higher diastolic blood pressure with positive ones.

**Figure 5:**
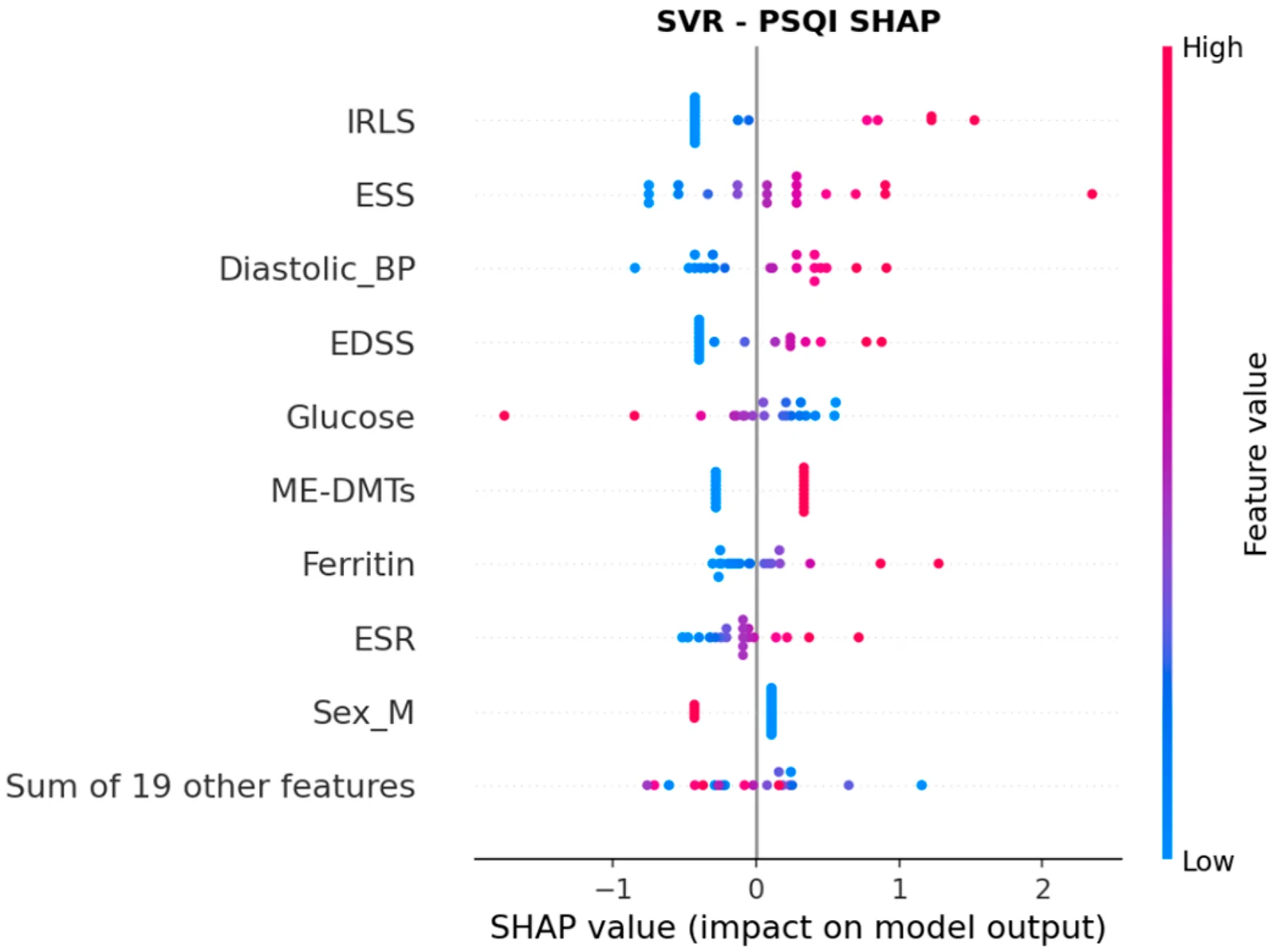
SHAP beeswarm summary plot for the SVR model. Features are ranked by mean absolute SHAP value. Positive values indicate a contribution toward higher predicted PSQI scores. Abbreviations: IRLS—International Restless Legs Syndrome Scale score; ESS—Epworth Sleepiness Scale score; Diastolic_BP—diastolic blood pressure; EDSS—Expanded Disability Status Scale score; ME-DMTs—moderate-efficacy disease-modifying therapies; ESR—erythrocyte sedimentation rate; Sex_M—male sex.

In the SVR model (Figure 5), IRLS and ESS scores were the top two predictors, with higher values associated with positive SHAP values, indicating a contribution toward higher predicted PSQI scores. Diastolic blood pressure and EDSS followed, showing the same direction of effect. Higher fasting glucose values were associated with negative SHAP values. ME-DMT treatment category, ferritin, ESR, and male sex were also identified among the top predictors, with higher ferritin and ESR values contributing toward higher predicted scores and male sex toward lower ones.

**Figure 6:**
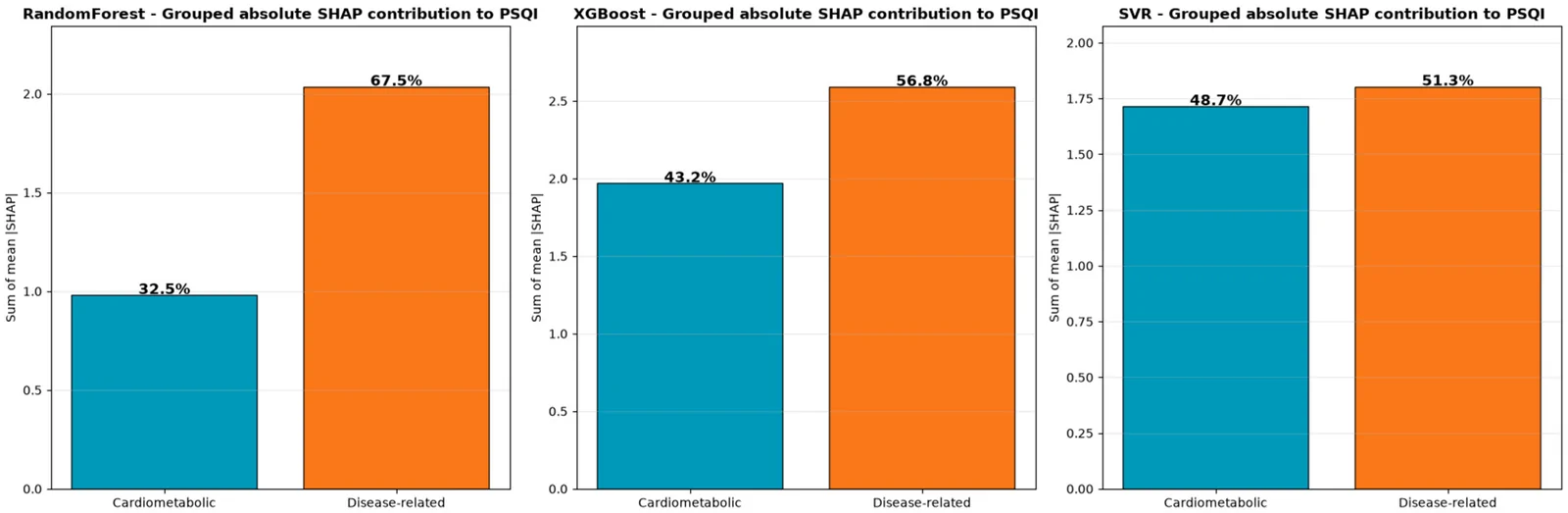
Grouped absolute SHAP contributions to predicted PSQI scores by predictor domain (disease-related and symptomatic vs. cardiometabolic and inflammatory) for Random Forest, XGBoost, and SVR models. Abbreviations: SHAP—SHapley Additive exPlanations; PSQI—Pittsburgh Sleep Quality Index; SVR—Support Vector Regression.

The aggregated SHAP analysis by predictor domain is presented in Figure 6. Across all three models, the disease-related and symptomatic domain accounted for a larger proportion of the total absolute SHAP contribution than the cardiometabolic and inflammatory domain: Random Forest (67.5% vs. 32.5%), XGBoost (56.8% vs. 43.2%), and SVR (51.3% vs. 48.7%).

## 4. Discussion

The present study suggests that machine learning models can model sleep quality in patients with MS, with XGBoost achieving the best performance (R^2^ = 0.451). Interpreting this value requires appropriate context: in traditional clinical regression, where biological and behavioral variability limit predictive power, R^2^ values in the range of 0.15 to 0.20 have been proposed as meaningful benchmarks [28]. Machine learning models applied to high-dimensional clinical datasets typically achieve higher predictive performance, with R^2^ values spanning approximately 0.42 to 0.75 depending on the algorithm [29]. The present model falls within this expected range, explaining 45.1% of the variance in PSQI scores. This is consistent with sleep disturbance in MS being associated with a complex interplay of neurological, metabolic, and inflammatory factors. The wide confidence intervals observed across all models reflect the limited sample size available for this analysis. Nonetheless, all three trained models substantially outperformed a reference model based on the mean PSQI value (test R^2^ = −0.059). Both tree-based models outperformed the linear SVR (test R^2^ 0.451 and 0.380 versus 0.336). Although this difference was not formally tested for statistical significance given the small test set, it is consistent with the possibility that some relationships between clinical parameters and sleep quality are more complex than a linear model can capture.

RLS is a neurological disorder characterized by an uncomfortable urge to move the legs that typically worsens at rest and improves with movement, particularly at bedtime, and it occurs more frequently in patients with neurological conditions such as MS across ethnic groups and geographic regions [30,31]. Higher RLS symptom severity, as captured by the IRLS score, was the strongest predictor of poor sleep quality across all three models, with greater severity consistently associated with higher predicted PSQI scores. This aligns with a recent study that identified RLS as a strong independent predictor of poor sleep quality in MS [32]. RLS represents a major contributor to impaired sleep quality, excessive daytime sleepiness, and fatigue [33,34], and in MS it has been associated in particular with prolonged sleep onset latency and more frequent nocturnal disturbances [34]. RLS is considerably more frequent in MS than in the general population, with a pooled prevalence of around 28% versus 8% in controls, and is more common in women [35]. This higher prevalence may have a structural basis, as pyramidal tract involvement has been identified as an independent risk factor for RLS in MS [36].

Across all three models, EDSS emerged as a primary predictor of sleep quality, with higher disability consistently associated with higher predicted PSQI scores. The literature, however, suggests that this relationship is neither strong nor direct. Several studies have reported only weak correlations between EDSS and PSQI scores [37], and the effect of disability on sleep appears to be largely indirect: in one cohort, the link between disability and poor sleep was mediated by depression, pain, and physical fatigue rather than acting directly [38]. Consistent with this, some studies have found that global EDSS does not independently predict sleep quality, with specific functional impairments, such as the visual functional system subscore, and comorbid depression emerging as the relevant predictors instead [39]. Taken together, these findings suggest that disability may contribute to poor sleep in MS mainly through co-occurring symptoms, which may explain why its effect is difficult to capture with simple bivariate analyses and underscores the value of multivariable approaches able to model these interacting determinants.

Excessive daytime sleepiness, a common daytime manifestation of disturbed sleep, was another consistent predictor in our analysis. ESS ranked among the top two predictors in the SVR model, where higher values contributed toward higher predicted PSQI scores, and featured prominently in the Random Forest and XGBoost models. Excessive daytime sleepiness has been reported in 40–45% of patients with MS [40], and its association with poorer sleep quality is well documented: a significant positive correlation between ESS and PSQI has been described in MS cohorts [12], and baseline ESS has been shown to independently predict poor sleep quality at one-year follow-up [41]. Comorbidities may further shape this relationship, as Paucke et al. reported that daytime sleepiness correlated with PSQI only in patients with associated conditions such as anemia, thyroid dysfunction, or depression, and not in those without [42].

Depression emerged as a prominent predictor of poor sleep quality in the Random Forest and XGBoost models, in line with what may represent a bidirectional relationship between depression and sleep disturbance in MS [14]. This association is not consistently observed, however: some studies have reported that depression does not remain significantly associated with poor sleep once other factors are accounted for, suggesting that the depression–sleep relationship in MS may be complex and influenced by additional variables [43]. Moreover, in the present study, depression was defined partly on the basis of ongoing antidepressant treatment, and several antidepressants directly influence sleep architecture. The observed contribution of depression to predicted PSQI scores may therefore partly reflect the pharmacological management of these patients rather than the mood disorder itself.

The treatment category also emerged as a relevant predictor, with ME-DMTs contributing consistently across all three models. A review addressing the impact of MS therapies on sleep concluded that interferon-beta and some symptomatic medications may worsen sleep quality, whereas higher-efficacy agents such as natalizumab may have a neutral or even favorable effect [44]. This pattern is consistent with our finding that ME-DMT treatment, rather than HE-DMT, was associated with higher predicted PSQI scores, suggesting that the observed effect may reflect the adverse-effect profile of specific agents rather than treatment efficacy per se. However, evidence in this area remains limited: few studies have examined the effect of individual DMTs on sleep quality [45], and, to our knowledge, none have directly compared different DMTs in this respect. This potential association should therefore be interpreted with caution.

Male sex emerged as a predictor only in the SVR model, where it contributed toward better predicted sleep quality, consistent with the poorer sleep reported among women with MS [46]. Given its emergence in a single model and the modest number of male participants, this finding remains preliminary.

Although the major predictors identified by our models were discussed separately, the present findings suggest that poor sleep quality in MS is unlikely to result from a single dominant factor. Instead, it appears to reflect the combined influence of several closely related clinical features. Patients with greater neurological disability often experience symptoms such as pain, reduced mobility, nocturia, and RLS, all of which may contribute to sleep fragmentation and poorer sleep quality [12]. In turn, chronic sleep disruption may worsen daytime sleepiness and depressive symptoms, creating a pattern in which disability, sleep impairment, and mood disturbance may be interrelated. Such interdependence between clinical domains would be expected to produce effects that are more complex than a linear model can capture, which may help explain why the tree-based models captured this pattern more effectively than the linear approach. This finding illustrates the use of interpretable machine learning as a complementary approach for exploring the multifactorial determinants underlying sleep disturbances in MS.

Cardiometabolic and inflammatory parameters appeared among the influential predictors in all three models, though their contributions were weaker and less coherent than those of the disease-related factors. 

Diastolic blood pressure represented the most physiologically plausible finding among these parameters, ranking among the leading predictors in both the XGBoost and SVR models, with higher values contributing toward poorer predicted sleep. This is consistent with studies linking elevated blood pressure to higher PSQI scores [47], and with polysomnographic evidence showing that reduced sleep efficiency, frequent arousals, and a higher apnea burden are associated with elevated nocturnal diastolic blood pressure and with a blunted day-to-night dipping profile [48].

The remaining cardiometabolic parameters showed less coherent patterns. Higher fasting glucose, larger waist circumference, and, in the Random Forest model, higher uric acid and triglycerides all carried negative SHAP values, contributing toward lower rather than higher predicted PSQI scores. These directions are inconsistent with previous reports. In MS, adiposity has received little attention, although higher BMI has been identified as a baseline predictor of poor sleep quality at one-year follow-up [40] and as a predictor of obstructive sleep apnea [49]. In the general population, larger waist circumference has likewise been associated with poorer sleep [50]. Given that the cohort was young (mean age 39.7 years), with few metabolic comorbidities and diabetes in only 3.5%, limiting the metabolic variance available to the models, and given the weak correlations between these variables and PSQI observed in the correlation matrix, these are more likely to reflect random variation than protective effects.

Among the inflammatory indices, ESR and ferritin featured only in the SVR model, where higher values contributed toward higher predicted PSQI scores. This direction is compatible with studies linking systemic inflammation to poorer sleep quality [51]. However, NLR appeared only in the Random Forest model, with higher values associated with negative SHAP values, and the systemic immune-inflammation index did not emerge in any model. A similar pattern has been described in other chronic inflammatory diseases: in a cohort of patients with rheumatoid arthritis, several metabolic indices correlated with PSQI scores in univariate analysis but lost significance once disease activity and inflammatory markers were accounted for, while NLR likewise failed to retain independent predictive value; the authors concluded that metabolic dysfunction may act on sleep indirectly, through systemic inflammation and disease activity, rather than directly [52]. The inflammatory indices therefore behaved inconsistently across models in our cohort, and did not reproduce the association reported in the wider literature.

The grouped SHAP analysis confirmed that, across all three models, the disease-related and symptomatic domain represented a higher percentage of the total absolute SHAP contribution, though the magnitude of this difference varied across models. Overall, the metabolic and inflammatory domain contributed less to sleep quality in MS than disease-related and symptomatic factors, and the associations seen here should be considered exploratory.

The limited contribution of cardiometabolic and inflammatory parameters may partly reflect the characteristics of our cohort, which included predominantly young patients with RRMS and a low burden of metabolic comorbidities. These findings do not exclude a role for cardiometabolic factors but suggest that their contribution may become more apparent in older patients, in those with greater comorbidity, or later in the disease course. Further studies in such populations are needed to clarify this.

The findings may also have clinical implications. RLS, which emerged as the strongest and most consistent predictor across all three models, remains frequently underrecognized in patients with MS despite the availability of effective pharmacological and non-pharmacological interventions [11]. Likewise, excessive daytime sleepiness and depressive symptoms can be assessed using brief, validated questionnaires that are easily incorporated into routine clinical practice. Together, these findings suggest that a simple symptom-oriented assessment focused on RLS, daytime sleepiness, and mood may help identify patients at increased risk of poor sleep quality during routine clinical care. Because poor sleep contributes to fatigue, cognitive dysfunction, and reduced health-related quality of life in MS [6,7,8,9], earlier recognition and targeted management of these symptoms may provide benefits that extend beyond sleep itself.

Several limitations should be acknowledged. First, the substantial performance gap between training (R^2^ = 0.864) and test (R^2^ = 0.451) in the XGBoost model indicates overfitting, reflecting the sample-size-to-predictor ratio: with 138 training cases and 28 features, the model may capture training-specific patterns rather than generalizable relationships. 

Second, the study was conducted at a single center, and the cohort consisted almost entirely of patients with RRMS and mild disability. Consequently, these predictive models cannot be reliably extrapolated to progressive MS phenotypes or to patients with severe neurological impairment.

Third, the cross-sectional design precludes causal inference regarding the identified associations. Because predictors and the outcome were measured at the same visit, the models reflect concurrent associations rather than prospective prediction.

Fourth, although medication use (including disease-modifying therapy category, gabapentinoids, baclofen, and antidepressant status) was incorporated into the models, the effects of individual agents, treatment dose, duration, adherence, and other medications with potential effects on sleep could not be fully accounted for. Residual confounding related to medication use therefore cannot be excluded. Several factors known to influence sleep quality in MS, including fatigue, pain, nocturia, anxiety, insomnia severity, and obstructive sleep apnea, were not assessed in the present study and could not be included as predictors.

Finally, sleep quality and related symptoms were assessed exclusively through questionnaires. As subjective instruments, these are susceptible to the influence of the respondent’s emotional and cognitive state at the time of completion, an inherent source of measurement variability that cannot be entirely eliminated. 

Taken together, these limitations, including the modest sample size, indicate that the present findings should be regarded as exploratory, warranting confirmation in larger, independent cohorts before clinical application can be considered.

## 5. Conclusions

This study demonstrates that machine learning, particularly XGBoost, can characterize sleep quality in MS, explaining approximately 45% of the variance in PSQI scores. Sleep impairment in this population was most strongly associated with RLS severity, neurological disability, and depression. Several cardiometabolic parameters also emerged among the influential predictors, although the direction of their contribution was not always consistent with physiological expectations, and systemic inflammatory indices did not contribute meaningfully. Disease-related and symptomatic factors therefore appear to outweigh cardiometabolic and inflammatory contributions to sleep quality in MS. These findings suggest the potential of interpretable machine learning to identify clinically relevant correlates of sleep quality, and highlight the need for validation in larger and more diverse MS cohorts.

## Figures and Tables

**Table 1:** Baseline demographic and clinical characteristics of the study participants.

Variable	Mean ± SD/n (%)
Demographics	
Age (years)	39.66 ± 11.86
Female	121 (69.9%)
Male	52 (30.1%)
Smoking (yes)	38 (22.2%)
Coffee consumption (yes)	108 (70.1%)
BMI (kg/m^2^)	25.59 ± 5.31
Clinical	
MS Form	
RRMS	156 (90.2%)
SPMS	10 (5.8%)
PPMS	7 (4.0%)
Disease duration (years)	10.84 ± 7.61
EDSS score	2.73 ± 1.76
Relapses	4.17 ± 3.23
Treatment	
HE-DMTs	100 (57.8%)
ME-DMTs	56 (32.4%)
Gabapentinoid (evening)	27 (15.6%)
Baclofen (evening)	14 (8.1%)
Sleep	
PSQI total score	6.06 ± 3.47
Poor sleepers	83 (48.0%)
ESS score	4.47 ± 3.43
RLS present	76 (43.9%)
Comorbidities	
Hypertension	22 (12.7%)
Dyslipidemia	30 (17.3%)
Diabetes	6 (3.5%)
Depression	21 (12.1%)
Laboratory	
Ferritin (ng/mL)	82.64 ± 97.48
SII	532.32 ± 352.01
NLR	2.14 ± 1.62
ESR (mm/h)	12.42 ± 9.27
Uric acid (mg/dL)	4.82 ± 1.38
HDL-cholesterol (mg/dL)	54.94 ± 14.59
Triglycerides (mg/dL)	92.69 ± 44.26
Fasting glucose (mg/dL)	91.37 ± 13.11

Abbreviations: BMI—Body Mass Index; MS—multiple sclerosis; RRMS—relapsing-remitting multiple sclerosis; SPMS—secondary progressive multiple sclerosis; PPMS—primary progressive multiple sclerosis; EDSS—Expanded Disability Status Scale; HE-DMTs—high-efficacy disease-modifying therapies; ME-DMTs—moderate-efficacy disease-modifying therapies; PSQI—Pittsburgh Sleep Quality Index; ESS—Epworth Sleepiness Scale; RLS—restless legs syndrome; SII—Systemic Immune-Inflammation Index; NLR—neutrophil-to-lymphocyte ratio; ESR—erythrocyte sedimentation rate; HDL—high-density lipoprotein.

**Table 2:** Model performance metrics for PSQI prediction.

Model	Train R^2^	Test R^2^ [95% CI]	Test MAE [95% CI]	Test RMSE [95% CI]
SVR (linear)	0.376	0.336 [0.078, 0.448]	2.322 [1.883, 2.759]	2.630 [2.158, 3.085]
Random Forest	0.734	0.380 [0.116, 0.528]	2.138 [1.694, 2.597]	2.542 [2.015, 3.057]
XGBoost	0.864	0.451 [0.145, 0.630]	1.956 [1.487, 2.485]	2.392 [1.784, 2.937]
Mean PSQI (reference)		−0.059	2.880	3.322

## Data Availability

The data presented in this study are available on request from the corresponding author. This restriction arises from privacy and ethical concerns regarding patient confidentiality.

## Supplementary Materials

The following supporting information can be downloaded at https://www.mdpi.com/article/10.3390/medsci14040483/s1, Figure S1: Correlation matrix of the predictor variables; Table S1: Missing data for each predictor variable.

## Abbreviations

The following abbreviations are used in this manuscript:

BMI	Body Mass Index
DMT	Disease-Modifying Therapy
EDSS	Expanded Disability Status Scale
ESR	Erythrocyte Sedimentation Rate
ESS	Epworth Sleepiness Scale
HDL	High-Density Lipoprotein
HE-DMT	High-Efficacy Disease-Modifying Therapy
IQR	Interquartile Range
IRLS	International Restless Legs Syndrome Scale
KNN	K-Nearest Neighbors
MAE	Mean Absolute Error
ME-DMT	Moderate-Efficacy Disease-Modifying Therapy
MS	Multiple Sclerosis
NLR	Neutrophil-to-Lymphocyte Ratio
PSQI	Pittsburgh Sleep Quality Index
R^2^	Coefficient of Determination
RLS	Restless Legs Syndrome
RMSE	Root Mean Squared Error
RRMS	Relapsing-Remitting Multiple Sclerosis
SD	Standard Deviation
SHAP	SHapley Additive exPlanations
SII	Systemic Immune-Inflammation Index
SVR	Support Vector Regression
XGBoost	eXtreme Gradient Boosting
