# Supplementary material for: Machine Learning-Based Prediction of Sleep Quality in Patients with Multiple Sclerosis
Source: Med Sci (Basel). 2026 Aug 14;14(4):483. doi: 10.3390/medsci14040483 (PMC13515246; doi:10.3390/medsci14040483)
Supplement: Supplementary file 1 [file medsci-14-00483-s001.zip › Supplementary Table S1.pdf]

Supplementary Table S1. Missing data for each predictor variable (n = 173)

Variable	Available (n)	Missing (n)	Missing (%)
**Disease-related and symptomatic**			
MS Form	173	0	0.0
EDSS	172	1	0.6
IRLS	173	0	0.0
Disease duration	172	1	0.6
Relapses	154	19	11.0
DMT category	156	17	9.8
ESS	173	0	0.0
Gabapentinoids (daytime)	173	0	0.0
Gabapentinoids (evening)	173	0	0.0
Baclofen (daytime)	172	1	0.6
Baclofen (evening)	172	1	0.6
Depression	173	0	0.0
**Cardiometabolic and inflammatory**			
Smoking	171	2	1.2
Coffee consumption	154	19	11.0
Sex	173	0	0.0
Waist circumference	172	1	0.6
BMI	170	3	1.7
Systolic BP	166	7	4.0
Diastolic BP	167	6	3.5
HDL-cholesterol	172	1	0.6
Uric acid	173	0	0.0
Triglycerides	171	2	1.2
Fasting glucose	170	3	1.7
Diabetes	173	0	0.0
Ferritin	160	13	7.5
ESR	173	0	0.0
NLR	173	0	0.0
SII	173	0	0.0

Abbreviations: EDSS – Expanded Disability Status Scale; IRLS – International Restless Legs Syndrome Scale; DMT – disease-modifying therapy; ESS – Epworth Sleepiness Scale; BMI – Body Mass Index; BP – blood pressure; HDL – high-density lipoprotein; ESR – erythrocyte sedimentation rate; NLR – neutrophil-to-lymphocyte ratio; SII – Systemic Immune-Inflammation Index.
